# Supplementary material for: Modeling glioblastoma heterogeneity as a dynamic network of cell states
Source: Mol Syst Biol. 2021 Sep 16;17(9):e10105. doi: 10.15252/msb.202010105 (PMC8444284; doi:10.15252/msb.202010105)
Supplement: Supplementary file 5 — Source Data for Figure 3 [file MSB-17-e10105-s001.zip › Figure3A_sourcedata/GSEA_3065/nosigdb_state6.GseaPreranked.1622718443993/NB_KI_CLUSTER4.html]

Details for gene set NB\_KI\_CLUSTER4[GSEA]

|  || Dataset | state6 |
| Phenotype | NoPhenotypeAvailable |
| Upregulated in class | na\_pos |
| GeneSet | NB\_KI\_CLUSTER4 |
| Enrichment Score (ES) | 0.4633009 |
| Normalized Enrichment Score (NES) | 1.959603 |
| Nominal p-value | 0.0 |
| FDR q-value | 0.0013007576 |
| FWER p-Value | 0.013 |
Table: GSEA Results Summary

  

Fig 1: Enrichment plot: NB\_KI\_CLUSTER4      
 Profile of the Running ES Score & Positions of GeneSet Members on the Rank Ordered List

  

| PROBE | GENE SYMBOL | GENE\_TITLE | RANK IN GENE LIST | RANK METRIC SCORE | RUNNING ES | CORE ENRICHMENT || 1 | SOX4 |  |  | 3 | 0.375 | 0.0495 | Yes |
| 2 | MYL6B |  |  | 83 | 0.223 | 0.0700 | Yes |
| 3 | GLCCI1 |  |  | 101 | 0.213 | 0.0964 | Yes |
| 4 | H3F3A |  |  | 104 | 0.212 | 0.1243 | Yes |
| 5 | SNRPN |  |  | 115 | 0.206 | 0.1505 | Yes |
| 6 | CIRBP |  |  | 151 | 0.190 | 0.1717 | Yes |
| 7 | TTC3 |  |  | 160 | 0.188 | 0.1957 | Yes |
| 8 | NLRP1 |  |  | 203 | 0.175 | 0.2142 | Yes |
| 9 | GNAS |  |  | 210 | 0.174 | 0.2366 | Yes |
| 10 | MARCKSL1 |  |  | 223 | 0.169 | 0.2576 | Yes |
| 11 | PTMS |  |  | 269 | 0.157 | 0.2733 | Yes |
| 12 | GTF2I |  |  | 284 | 0.154 | 0.2921 | Yes |
| 13 | FXYD6 |  |  | 387 | 0.138 | 0.2987 | Yes |
| 14 | CENPV |  |  | 404 | 0.135 | 0.3149 | Yes |
| 15 | MCRIP1 |  |  | 405 | 0.135 | 0.3329 | Yes |
| 16 | YWHAH |  |  | 449 | 0.128 | 0.3450 | Yes |
| 17 | DPYSL3 |  |  | 464 | 0.127 | 0.3602 | Yes |
| 18 | YWHAQ |  |  | 478 | 0.125 | 0.3754 | Yes |
| 19 | H3F3B |  |  | 514 | 0.122 | 0.3875 | Yes |
| 20 | GDI1 |  |  | 562 | 0.117 | 0.3976 | Yes |
| 21 | BEX3 |  |  | 637 | 0.111 | 0.4039 | Yes |
| 22 | ATP6V0E2 |  |  | 676 | 0.108 | 0.4139 | Yes |
| 23 | SUMO2 |  |  | 710 | 0.105 | 0.4240 | Yes |
| 24 | RBP1 |  |  | 788 | 0.099 | 0.4283 | Yes |
| 25 | TUBB2B |  |  | 795 | 0.099 | 0.4407 | Yes |
| 26 | CDK6 |  |  | 807 | 0.097 | 0.4524 | Yes |
| 27 | ICA1 |  |  | 1102 | 0.080 | 0.4293 | Yes |
| 28 | TERF2IP |  |  | 1272 | 0.073 | 0.4196 | Yes |
| 29 | STMN3 |  |  | 1279 | 0.072 | 0.4285 | Yes |
| 30 | CCND1 |  |  | 1317 | 0.071 | 0.4336 | Yes |
| 31 | CKB |  |  | 1359 | 0.069 | 0.4380 | Yes |
| 32 | QDPR |  |  | 1420 | 0.067 | 0.4400 | Yes |
| 33 | PRKAR2B |  |  | 1482 | 0.065 | 0.4416 | Yes |
| 34 | FSCN1 |  |  | 1498 | 0.064 | 0.4484 | Yes |
| 35 | PEG10 |  |  | 1556 | 0.062 | 0.4501 | Yes |
| 36 | RUFY3 |  |  | 1615 | 0.060 | 0.4514 | Yes |
| 37 | ATCAY |  |  | 1635 | 0.059 | 0.4570 | Yes |
| 38 | STMN1 |  |  | 1649 | 0.058 | 0.4633 | Yes |
| 39 | TCAF1 |  |  | 1774 | 0.054 | 0.4563 | No |
| 40 | ZC3H13 |  |  | 1968 | 0.049 | 0.4406 | No |
| 41 | OCIAD2 |  |  | 2061 | 0.046 | 0.4361 | No |
| 42 | JPT1 |  |  | 2094 | 0.045 | 0.4384 | No |
| 43 | VAMP2 |  |  | 2100 | 0.045 | 0.4438 | No |
| 44 | TUBA1A |  |  | 2135 | 0.044 | 0.4457 | No |
| 45 | SLC38A1 |  |  | 2301 | 0.039 | 0.4318 | No |
| 46 | MLLT11 |  |  | 2335 | 0.038 | 0.4331 | No |
| 47 | CALM1 |  |  | 2795 | 0.027 | 0.3839 | No |
| 48 | CBX1 |  |  | 2863 | 0.025 | 0.3795 | No |
| 49 | MAP1B |  |  | 2893 | 0.024 | 0.3794 | No |
| 50 | MYEF2 |  |  | 3067 | 0.021 | 0.3622 | No |
| 51 | ATAT1 |  |  | 3237 | 0.017 | 0.3451 | No |
| 52 | TRIM2 |  |  | 3259 | 0.016 | 0.3448 | No |
| 53 | STXBP1 |  |  | 3310 | 0.015 | 0.3411 | No |
| 54 | PHF14 |  |  | 3551 | 0.010 | 0.3149 | No |
| 55 | NREP |  |  | 3707 | 0.007 | 0.2980 | No |
| 56 | TSPAN13 |  |  | 3733 | 0.006 | 0.2959 | No |
| 57 | MTSS1 |  |  | 3804 | 0.005 | 0.2885 | No |
| 58 | APLP1 |  |  | 3813 | 0.004 | 0.2881 | No |
| 59 | C3orf14 |  |  | 3893 | 0.003 | 0.2794 | No |
| 60 | YWHAE |  |  | 3963 | 0.001 | 0.2717 | No |
| 61 | SYT11 |  |  | 4256 | -0.004 | 0.2387 | No |
| 62 | MDK |  |  | 4311 | -0.005 | 0.2332 | No |
| 63 | CXADR |  |  | 4321 | -0.005 | 0.2328 | No |
| 64 | KIF3A |  |  | 4823 | -0.015 | 0.1773 | No |
| 65 | GDAP1 |  |  | 4904 | -0.017 | 0.1703 | No |
| 66 | CALM2 |  |  | 5062 | -0.020 | 0.1549 | No |
| 67 | NELFCD |  |  | 5271 | -0.024 | 0.1341 | No |
| 68 | HDAC2 |  |  | 5329 | -0.025 | 0.1309 | No |
| 69 | MIAT |  |  | 5393 | -0.026 | 0.1271 | No |
| 70 | NME1 |  |  | 5479 | -0.028 | 0.1211 | No |
| 71 | ENO2 |  |  | 5889 | -0.036 | 0.0789 | No |
| 72 | RBFOX2 |  |  | 5896 | -0.036 | 0.0829 | No |
| 73 | TMEM14A |  |  | 5961 | -0.037 | 0.0805 | No |
| 74 | NSD3 |  |  | 6057 | -0.039 | 0.0748 | No |
| 75 | RIC3 |  |  | 6255 | -0.044 | 0.0580 | No |
| 76 | PEBP1 |  |  | 6561 | -0.052 | 0.0298 | No |
| 77 | HSP90AB1 |  |  | 6619 | -0.053 | 0.0304 | No |
| 78 | TUBB |  |  | 6820 | -0.059 | 0.0153 | No |
| 79 | TUBB2A |  |  | 7078 | -0.067 | -0.0053 | No |
| 80 | COX6A1 |  |  | 7089 | -0.067 | 0.0025 | No |
| 81 | ZNF704 |  |  | 7300 | -0.074 | -0.0118 | No |
| 82 | CFAP20 |  |  | 7355 | -0.076 | -0.0079 | No |
| 83 | KIF21A |  |  | 7417 | -0.078 | -0.0045 | No |
| 84 | UCHL1 |  |  | 7848 | -0.098 | -0.0408 | No |
| 85 | ATP1B1 |  |  | 8091 | -0.113 | -0.0536 | No |
| 86 | SEC11C |  |  | 8166 | -0.118 | -0.0464 | No |
| 87 | CADM1 |  |  | 8282 | -0.130 | -0.0423 | No |
| 88 | ATP6V0A1 |  |  | 8592 | -0.187 | -0.0530 | No |
| 89 | LUC7L3 |  |  | 8631 | -0.203 | -0.0303 | No |
| 90 | MEG3 |  |  | 8786 | -0.370 | 0.0011 | No |
Table: GSEA details [plain text format]

  

Fig 2: NB\_KI\_CLUSTER4: Random ES distribution      
 Gene set null distribution of ES for **NB\_KI\_CLUSTER4**
